# Challenges of diagnosis of COVID-19 in trauma patients: A case series

**DOI:** 10.1177/1460408620950602

**Published:** 2021-07

**Authors:** Golnar Sabetian, Farnia Feiz, Alireza Shakibafard, Hossein Abdolrahimzadeh Fard, Sepideh Sefidbakht, Seyed Hamed Jafari, HamidReza Abbasi, Masoomeh Zare, Amir Roudgari, Farid Zand, Mansoor Masjedi, Shahram Paydar

**Affiliations:** 1 Trauma Research Center, Shiraz University of Medical Sciences, Shiraz, Iran; 2 Department of Radiology, Weill Cornell Medical College, New York, NY, USA; 3 Department of Radiology, Shiraz University of Medical Sciences, Shiraz, Iran; 4 Medical Imaging Research Center, Shiraz University of Medical Sciences, Shiraz, Iran; 5 Anesthesiology and Critical Care Research Center, Shiraz University of Medical Sciences, Shiraz, Iran

**Keywords:** COVID-19, SARS-CoV-2, thoracic injuries, trauma, X-ray CT scans

## Abstract

**Background:**

Diagnosis of COVID-19 can be challenging in trauma patients, especially those with chest trauma and lung contusion.

**Methods:**

We present a case series of patients from February and March 2020 who were admitted to our trauma center at Rajaee Hospital Trauma Center, in Shiraz, Iran and had positive SARS-CoV-2 PCR test or chest CT scan suggestive of COVID-19 and were admitted to the specific ICU for COVID-19.

**Results:**

Eight COVID-19 patients (6 male) with mean age of 40 (SD = 16.3) years old, were presented. All patients were cases of trauma injuries, with multiple injuries including chest trauma and lung contusion, admitted to our trauma center for management of their injuries, but they were diagnosed with COVID-19 as well. Two of them had coinfection of influenza type-B and SARS-CoV-2. All patients were treated for COVID-19 and three of them died; the rest were discharged from hospital.

**Conclusion:**

Since PCR for SARS-CoV-2 is not always sensitive enough to confirm the cause of pneumonia, chest CT manifestations can be helpful, though, they are not always differentiable from lung contusion. Therefore, both the CT scan and the clinical and paraclinical presentation and course of improvement can be beneficial in diagnosing COVID-19 in the trauma setting.

## Introduction

In December 2020, an outbreak of a new coronavirus (SARS-CoV-2) causing Corona Virus Disease -19 (COVID-19) was reported by the World Health Organization (WHO). As of April 19, 2020, more than two million cases of COVID-19 and 150,000 deaths has been reported globally.^1^ Health authorities in Iran reported the first cases of COVID-19 to WHO on February 20,^2^ which increased to 80,868 confirmed cases and 5031 deaths by April 19, 2020.

COVID-19 patients can be asymptomatic or have symptoms of respiratory tract infection and manifestations of viral pneumonia on their chest CT scans; however, assessing these symptoms is not always feasible in situations such as trauma patients. While patients who present to trauma centers might be symptomatic or asymptomatic cases of COVID-19, diagnosis of this condition among trauma patients is challenging, which needs careful evaluation of the symptoms, clinical course, and chest CT findings.

So far, there are some studies on the clinical symptoms, characteristics, imaging, and outcome of COVID-19 patients, but reports of the trauma patients who also have SARS-CoV-2 infection are not available. In this study, we describe the clinical characteristics, imaging findings, and short term outcome of eight trauma patients who were diagnosed with COVID-19 and three suspicious cases who were then confirmed as negative COVID-19 after detailed evaluation, and discuss the challenges of diagnosing COVID-19 among trauma patients. All patients presented to Shahid Rajaee University Trauma hospital, a specialized level I trauma center in Shiraz, South of Iran.

## Methods

### Case finding and data collection

This retrospective case series study was approved by the Ethics Committee of Shiraz University of Medical Sciences (IRB code: IR.SUMS.REC.1399.123). All patients were enrolled in February and March 2020, and the patients or their next of kin were contacted, and informed consent was obtained.

All patients who had a positive SARS-CoV-2 reverse-transcription polymerase chain reaction (RT-PCR) test or a CT scan suggestive of COVID-19 pneumonia were included. Unidentified patients’ data, including medical reports, lab results, and imaging, were collected through electronic medical record review using the ID number of cases. Data of patients’ clinical course was obtained through intensivist’s and physicians’ documents who were involved in the management of patients.

### COVID-19 tests, diagnosis and treatment

A “confirmed” diagnosis was defined as those who had positive RT-PCR results according to samples taken from nasopharyngeal, oropharyngeal, or endotracheal tube aspirate. Detection kit for Novel Coronavirus 2019 (2019-nCoV) RNA (Fluorescent PCR) produced by DAAN Gene Co, Ltd of Sun Yat-sen University, China, was used. “Probable” cases were defined as patients who were highly suspicious for COVID-19 according to the high-resolution chest CT scan showing peripheral ground-glass opacity (GGO) with or without consolidation,^3,4^ with negative RT-PCR results with no other etiology that explained the CT findings and clinical presentation.^5,6^

The COVID-19 treatment protocol in our center, based on national guidelines, was Hydroxychloroquine: 400 mg every 12 hours on day 1, then 400 mg daily orally for 5 days, plus 20 mg of intravenous dexamethasone for 5 days, then 10 mg for 5 days, was administered to critically ill cases with Acute Respiratory Distress Syndrome (ARDS). We started empirical antibiotic therapy based on the susceptibility pattern of the pathogen when there was a clinical suspicion of bacterial pneumonia superinfection.^7^ Data analysis was not applicable for this study due to the descriptive nature of the data presented in our case series.

## Results

We present eight trauma patients (six male) with COVID-19, aged 18–62 years (mean 40 SD 16.3). All patients were admitted to a specific COVID-19 ICU and all except one were mechanically ventilated with a duration of 2–28 days. Coinfection of SARS-CoV-2 and influenza type B was detected in two patients; seven patients had leukocytosis, and six had different stages of lymphopenia. Table 1 demonstrates the clinical characteristics, presentation and outcomes, and Table 2 summarizes the lab results of the patients. A detailed table of laboratory data findings is provided in the supplemental material.

**Table 1.** Characteristics, presentation, complications and short term outcome of 8 COVID-19 patients and 3 non-COVID-19 patients.

	Case 1	Case 2	Case 3	Case 4	Case 5	Case 6	Case 7	Case 8	Case 9	Case 10	Case 11
Age and sex	43 y/o m	40 y/o m	58 y/o f	54 y/o m	62 y/o m	19 y/o m	26 y/o f	18 y/o m	19 y/o m	17 y/o m	20 y/o m
medical background	COPD, Hepatitis C, IV drug use	Substance use disorder	Asthma, DM, hyperthyroidism COPD	HTN, DM, COPD	HTN	None	Opioid use disorder	None	Substance use disorder (methadone) history of suicide attempt	None	DM
RT-PCR	+	+	+	_	_	+	_	+	_	_	_
COVID-19 related symptoms:											
Respiratory	respiratory distress	respiratory distress	Cough, dyspnea, chest pain	Respiratory distress	Respiratory distress	Respiratory distress	Cough, dyspnea	Respiratory distress	Dyspnea	Dyspnea	Dyspnea
Non-respiratory	Fever	Fever, body pain	Fever, body pain, diarrhea	Fever, body pain, diarrhea	Fever	Fever, body pain	Fever, body pain	Fever	Fever	Fever	Fever
Complications related to COVID-19											
ARDS	Yes	Yes	Yes	Yes	No	No	No	No	Yes	No	No
Arrhythmia	Yes	No	Yes	No	No	No	No	No	No	No	No
AKI	Yes	Yes	No	Yes	No	No	No	No	No	No	No
RRT	CRRT	No	No	CRRT	No	No	No	No	No	No	No
Duration of mechanical ventilation (days)	13	10	28	23	15	5	Not intubated	2	6	15	12
Outcome	Death	Death	Death	Discharged with tracheostomy	Discharged with tracheostomy	Discharged	Discharged	Discharged	Discharged	Discharged with tracheostomy	Discharged

COPD: chronic obstructive pulmonary disease, IV: intra-venous; DM: diabetes mellitus; HTN: hypertension, RT-PCR: reverse transcription polymerase chain reaction for SARS-CoV-2; ARDS: acute respiratory distress syndrome; AKI: acute kidney injury; RRT: renal replacement therapy; CRRT: chronic renal replacement therapy.

**Table 2.** Laboratory results of 8 COVID-19 and 3 non-COVID19 cases during their hospital course.

Lab tests	Case 1	Case 2	Case 3	Case 4	Case 5	Case 6	Case 7	Case 8	Case9	Case 10	Case 11
Lowest blood oxygen levels:											
PaO2 (mmHg)	58	45	42	30	25.9	35	39.9	49.9	41.5	65	55.2
SaO2 (%)	91%	70%	78%	56.4%	47%	64%	69.3%	82.4%	74.2%	71.3%	88%
Worst total WBC count (×10^9^ cells/L) during hospital course	15.7	2.7	26.9	18.6	24.3	29.3	16	21	21.8	22	13.5
Absolute neutrophil counts (×10^9^ cells/L, fraction %)	13.7 (87.8%)	1.9 (70%)	2.5 (91.7%)	15.6 (84%)	20.2 (83%)	25.2 (86%)	8.3 (52%)	16.2 (77%)	18.9 (87.5%)	19.1 (87%)	1.1 (84.1%)
Lowest absolute lymphocyte count (cells/ml, fraction %)	847 (5.4%)	192 (6%)	1760 (10.3%)	884 (8.5%)	1254 (6.6%)	1172 (4%)	1434 (16.3%)	2520 (12%)	1240 (20.9%)	1056 (4.8%)	1067 (11%)
CRP mg/L	150	80	89	56	N/A	92	79	60	80	99	48
ESR mm/hour	42	25	77	83	N/A	68	39	35	81	81	70
Procalcitonin	N/A	N/A	N/A	1.39	N/A	1.69	N/A	014	0.78	0.5	4.29
LDH (U/L)	1009	800	977	550	600	780	884	450	N/A	N/A	1433
D-dimer (µg/L)	1500	1100	850	3162	1500	2747	N/A	1718	4077	3062	N/A
Troponin level (ng/mL)	N/A	N/A	78	449	<1.5	N/A	N/A	N/A	N/A	1035	N/A
Highest BUN level (mg/dL)	101	100	19	56	34	20	7	14	11	16	31
Highest creatinine (mg/dL)	4.1	4	1.5	1.84	1.24	1.17	0.8	1.3	1.2	0.8	0.9
Highest SGOT level (U/L)	70	80	66	126	121	216	48	23	77	81	234
Highest SGPT level (U/L)	46	68	55	12	24	108	27	31	42	56	175

PaO2: partial pressure of oxygen; SaO_2_: oxygen saturation; WBC: white blood cells; CRP: C-reactive protein; ESR: erythrocyte sedimentation rate; LDH: lactate dehydrogenase; BUN: blood urea nitrogen; SGOT: serum glutamic-oxaloacetic transaminase; SGPT: serum glutamic-pyruvic transaminase.

### Case presentation

#### Case 1

**Figure 1.**
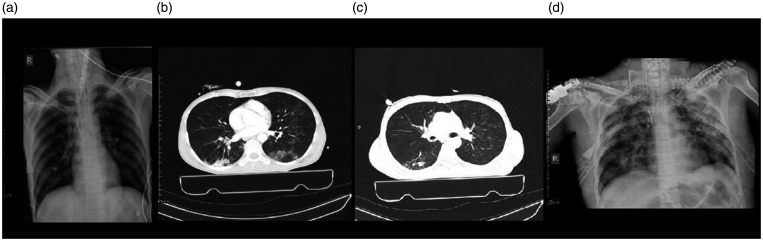
Chest imaging of a 43-year-old man, a case of coinfection of COVID-19, and influenza type B admitted in trauma center due to multiple injuries. 1 A: chest X-ray showed faint patchy GGO in the peripheral aspect of both lungs. 1B: an axial image of the CT scan showed wedge shape consolidation in the posterior of the right lower lobe. Patchy GGO is seen in the left lung with posterior predominance. 1 C: nodular consolidation is demonstrated in the posterior of the right lower lung. Also, patchy round shape GGO is seen in both lungs with left side predominance. 1 D: chest X-ray showed diffuse patchy consolidation in both lung fields.

A 43-year-old male involved in a motor car accident (MCA) suffered a ruptured eye globe, head injury, fracture of facial bones, brain contusion, C7 vertebrae, skull base, and femoral fractures. In the initial ED evaluation, he had leukocytosis and lymphopenia, but no fever or dyspnea. Chest X-Ray (CXR) and CT scan showed faint patchy ground-glass opacity (GGO) in the peripheral aspect of both lungs with nodular consolidation (Figure 1(a) and (b)). The patient was emergently brought to the operating room (OR) for management of the eye injury and femoral fracture. Due to the severe facial trauma, the patient was intubated for airway protection, and tracheostomy performed after one day; after one week, whilst planning to wean from the ventilator, he developed fever and respiratory distress and worsening lung involvement on the CXR and CT (Figure 1(c) and (d)). PCR tests were positive for COVID-19 and influenza type B. Despite receiving COVID-19 and influenza treatment (Oseltamivir), the patient’s condition deteriorated within a few days; he developed respiratory and acute renal failure and despite continuous renal replacement therapy (CRRT) died from sudden arrhythmia and cardiac arrest.

#### Case 2

**Figure 2.**
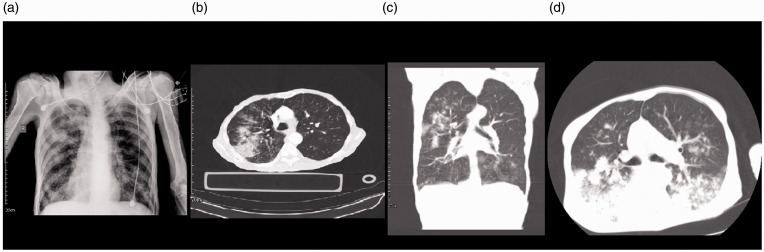
Chest imaging of a 40-year-old male with COVID-19. 2 A: Chest X-Ray showed multiple round shape opacities in both lungs more on the right side. 2B: patchy consolidation and GGO is seen in the right lung. 2 C: coronal reconstruction of chest CT showed nodular consolidation and GGO in the right upper lobe and left lower lobe. 2 D: an axial image of the chest CT scan demonstrated multiple round shape consolidations and GGO with posterior predominance. Also, diffused GGO in the posterior aspect of both lungs is seen.

A 40 year old male presented following a same level fall with minor injuries; however, the patient was febrile, dyspneic, and had body pain. CXR and CT scan showed multiple round shape opacities in both lungs and patchy consolidation and GGO in the right lung. (Figure 2(a) and (b)). RT-PCR for COVID-19 was positive, and since they did not need further management at the trauma center, he was transferred to a special hospital for COVID-19 cases. Despite receiving treatment, the patient developed renal failure, respiratory distress (Figure 2(c) and (d)) and died due to ARDS and multi-organ failure.

#### Case 3

**Figure 3.**
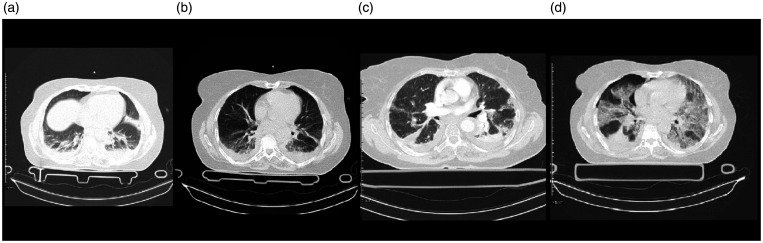
Chest imaging of a 58-year-old female with chest trauma and coinfection of Influenza type B and SARS-CoV-2. 3 A: multiple linear atelectasis and collapse consolidation are seen in the posterior of both lower lobes and lingula segment of left upper lobe. Mild pleural effusion is seen bilaterally. 3B: collapse consolidation and GGO are seen in the posterior of both lower lobes. Mild pleural effusion is seen bilaterally. 3 C: There are patchy GGO and consolidation in the peripheral aspect of both lungs more in posterior parts associated with bilateral mild pleural effusion. 3 D: mild pneumothorax is seen on the right side. Diffuse GGO and crazy paving pattern are seen in both lungs more on the left side. A wedge-shaped consolidation is seen in the posterior of the right lung. Minimal pleural effusion is seen bilaterally.

A 58-year-old female injured after a car rollover accident suffered lung contusion, left hemothorax (Figure 3(b)), and fractures of the radius and T8-T10 vertebrae. The initial trauma chest CT scan (Figure 3(a) and (b)) showed collapse, consolidation and pleural effusion. The patient was not hypoxic or dyspneic and was admitted to ICU for the management of trauma. After 10 days, she became febrile and hypoxic and was intubated. As the second chest CT shows (Figure 3(c) and (d)), she had diffuse and patchy GGO, crazy paving pattern, and consolidation in the peripheral aspect of both lungs. PCR tests came back positive for both influenza type B and SARS-CoV-2. Despite receiving treatment, the patient’s condition worsened, progressed to ARDS, and died due to severe ARDS and sudden cardiac arrhythmia.

#### Case 4

**Figure 4.**
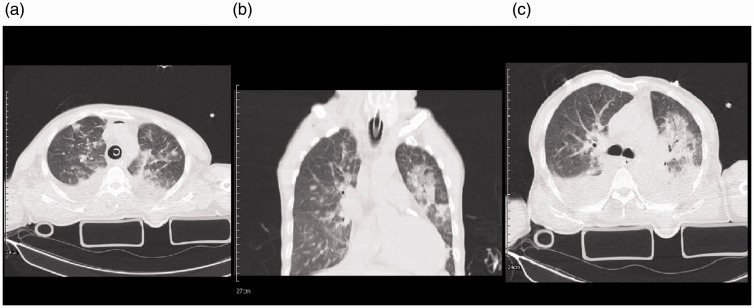
Chest imaging of a 54-year-old male with multiple trauma, including chest trauma, and SARS-CoV-2 infection. 4 A: interlobular septal thickening and patchy GGO and consolidation are seen in both lungs with associated bilateral pleural effusion. 4B: coronal reconstruction of chest CT showed bilateral patchy GGO and consolidation with septal thickening more on the left side. 4 C: an axial image of the chest CT scan in the level of carina showed relatively diffuse GGO and consolidation with septal thickening in the left side. Mild pleural effusion is seen bilaterally.

A 54-year-old male injured in a motorcycle-car accident event causing brain and lung contusions. The initial trauma chest CT showed patchy GGO and consolidation in both lungs with associated bilateral pleural effusion (Figure 4(a)). Blood tests revealed an increased leukocytosis. After a few days, the patient had a fever, symptoms of respiratory failure, and lymphopenia, and was intubated. He also had an acute renal injury in the course of hospital admission and underwent CRRT. Two SARS-CoV-2 PCR tests, and other respiratory workups were negative. However, based on the chest CT, the clinical course of the patient, and infectious diseases specialist consult, the patient was considered as probable COVID-19 case and hydroxychloroquine with steroid started. A tracheostomy was performed for respiratory management, and after recovering from renal and respiratory failure, he was discharged home with a tracheostomy.

#### Case 5

**Figure 5.**
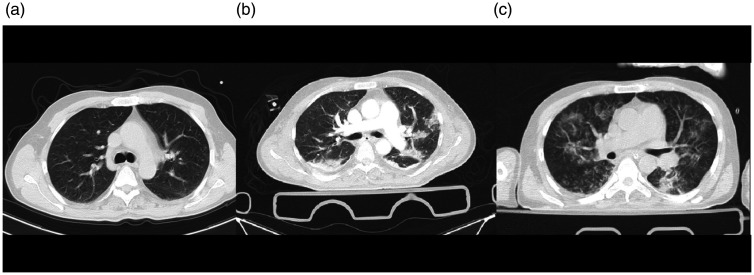
Chest CT scans of a 62-year-old man admitted to the trauma center and diagnosed with COVID-19. 5 A: an initial axial image of the chest CT scan in the level of carina showed normal findings. 5B: an axial image of the chest CT scan below the level of carina demonstrated patchy peripheral GGO and consolidation more in the posterior aspect. 5 C: multifocal patchy GGO is seen in both lungs.

A 62-year-old male with traumatic brain injury following an MCA was transferred to ICU for further management; the initial chest CT scan obtained at the time of hospital admission was normal (Figure 5(a)). After two days, the patient began with fever and dyspnea and was intubated. The blood tests showed leukocytosis, and repeat chest CT revealed patchy peripheral GGO and consolidation (Figure 5(b) and (c)). COVID-19 PCR tests were negative. However, based on the CT scan and infectious diseases specialist’s judgment, he was considered as a case of COVID-19, and treatment started including tracheostomy. After improvement of symptoms the patient was discharged home with a tracheostomy.

**Figure 6.**
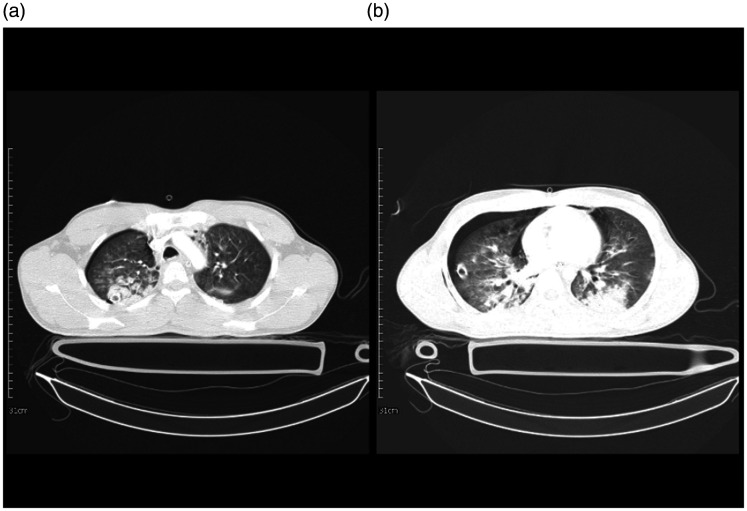
A 19-year-old man with multiple trauma and lung injury and a case of COVID-19. 6 A: an axial image of the chest CT scan in the level of aortic arch showed mild pneumothorax on the right side. There are multiple round shape GGO and consolidation in both upper lobes with right-side predominance. Also, minimal pneumomediastinum is noted. 6B: evidence of pneumomediastinum and right-sided pneumothorax with a chest tube is seen. Also, consolidation in the posterior aspect of both lungs associated with faint GGO is seen.

#### Case 6

A 19-year-old male was involved in a motorcycle accident and suffered brain contusions, fractures of left clavicle and zygomatic bone, and a right-side pneumothorax. The patient was febrile, had leukocytosis and lymphopenia on the day of admission. CT Figure 6(a) and (b) showed the right sided pneumothorax and a small pneumomediastinum as well as bilateral GGO. PCR for SARS-CoV-2 was positive. He was intubated due to respiratory distress and treatment started for him. The patient was extubated after 5 days and discharged home.

#### Case 7

**Figure 7.**
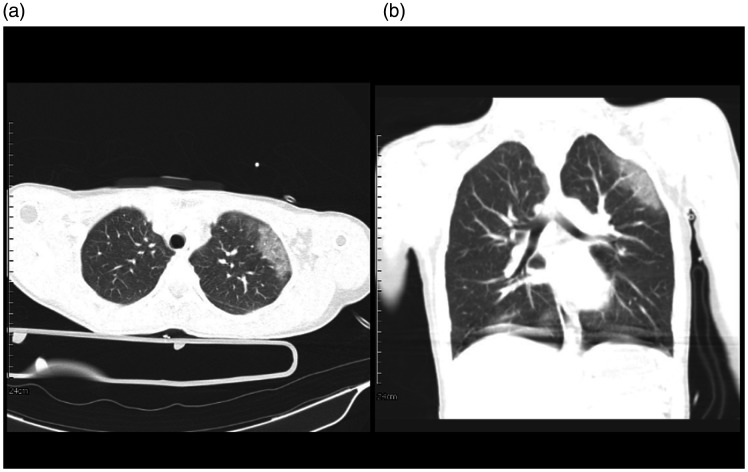
A 26-year-old female, positive case of COVID-19 in the trauma center. 7 A: Axial view, GGO is seen in the upper lobe of the left lung. 7B: coronal reconstruction of chest CT shows GGO on the left side.

A 26-year-old female, with a history of a substance use disorder, presented with fracture of C2 vertebrae, pelvic and sacrum due to a motor-vehicle collision. She was febrile and dyspneic, and the initial lab data showed leukocytosis. There was no evidence of chest trauma, but the initial trauma chest CT (Figure 7(a) and (b)) showed GGO suggestive of viral pneumonia. Although the PCR for COVID-19 was negative, based on the symptoms and CT manifestations, and negative results of other respiratory infection workup, she was considered as a probable case of COVID-19 and treatment started. She recovered and was discharged home on day 5 of admission.

#### Case 8

**Figure 8.**
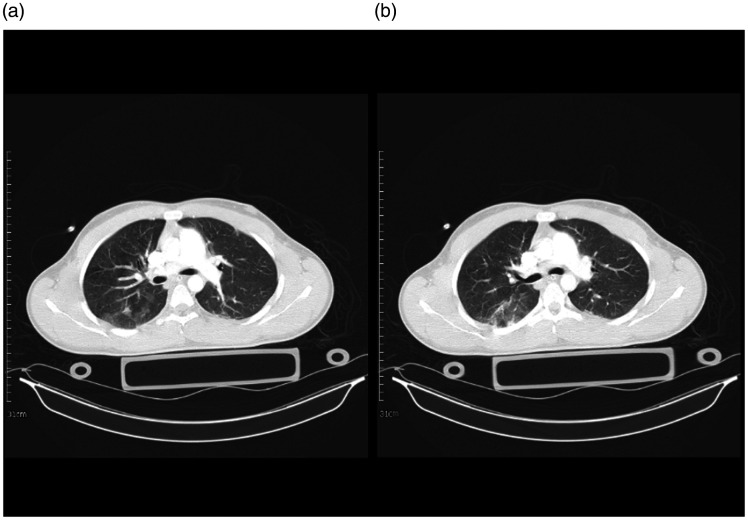
An 18-year-old male, trauma patient, and a positive case of COVID-19. 8 A: Multiple GGO in the right lung. 8B: GGO in the right lung and minimal pleura thickening in the left side.

An 18-year-old male was admitted with epidural hematoma and base of skull fracture after a fall. He was intubated due to decreased level of consciousness. He also had fever and leukocytosis on presentation, so PCR for SARS-CoV-2 was sent, and the result was positive. Chest CT showed GGO and pleural thickening (Figure 8(a) and (b)). He received treatment for COVID-19. He recovered from the infection and traumatic injury and was discharged home.

### Non-COVID-19 cases

The following 3 cases were initially suspected to be COVID-19, but based on the rapid resolution of chest CT and clinical improvement; they were diagnosed with other diseases.

#### Case 9

**Figure 9.**
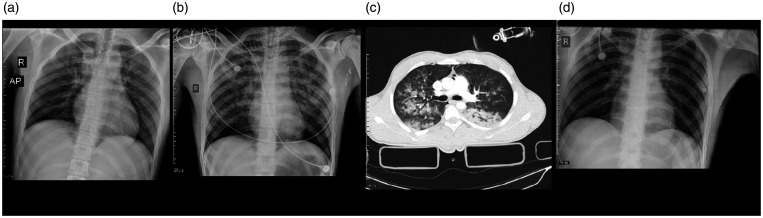
A 19-year-old male admitted to the trauma ICU who developed respiratory distress, which was suspicious for COVID-19, but with significant improvement, COVID-19 was ruled out. 9 A: CXR showed no abnormal opacity. 9B: CXR showed multiple round shape GGO and consolidations in both lung fields. 9 C: An axial image of the chest CT scan below the level of carina demonstrated diffuse round shape GGO and consolidation in both lungs with confluent consolidation in the left lower lobe. The radiological findings are typical for COVID-19 pneumonia. Also, evidence of pneumomediastinum is noted. 9 D: CXR showed normal findings with a complete resolution of opacities.

A 19-year-old male presented after a motorcycle-car accident with a fracture of the femur; the initial CXR was normal (Figure 9(a)). The patient had femoral surgery on day 2, and transferred to a ward after that, but after a few hours, developed severe respiratory distress, was intubated and transferred to ICU (Figure 9(b) and (c)). The patient had a fever, leukocytosis, and chest CT scan was suggestive of COVID-19 and so he was considered as a suspicious case of COVID-19 with a negative PCR test, however, after 2 days his condition improved significantly, and CXR was again normal (Figure 9(d)). The differential diagnosis for this patient could be lung contusion, lung emboli, or other types of viral pneumonia. After 6 days, the patient was extubated and discharged home. We believed this patient was not a case of COVID-19 based on the clinical improvement and resolution of chest CT scan within 2 days.

#### Case 10

**Figure 10.**
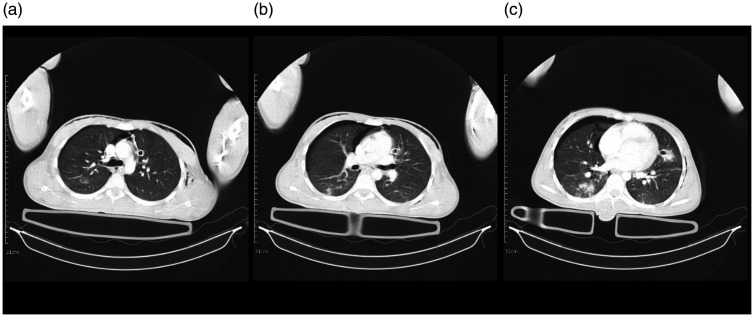
A 17-year-old man with multiple trauma including chest trauma, and a suspicious case of COVID-19 admitted in the ICU; however, COVID-19 was ruled out. 10 A: an axial image of the chest CT scan in the level of carina showed chest wall emphysema and mild right-sided pneumothorax. There is a small focus of GGO in the posterior of the right upper lobe. 10B: an axial image of the chest CT scan showed chest wall emphysema and mild right-sided pneumothorax. A small nodular infiltration is seen posterior of the right upper lobe. 10 C: minimal chest wall emphysema and right-sided pneumothorax are seen. There is a small focus of consolidation in the posterior of RLL and faint GGO in the posterior of the left lower lobe. A chest tube with adjacent infiltration is seen medial to the left upper lobe.

A 17-year-old male brought to the ED with epidural hematoma, a right pneumothorax and lung contusion (Figure 10(a)), and base of skull fracture after a motorcycle-car accident. The patient was intubated and transferred to ICU. On day 7 of admission, we found a decrease in oxygen saturation and fever in the daily assessment. CT scan (Figure 10(b)) showed a small focus of consolidation in the posterior of the right lower lobe and faint GGO. The patient was suspicious of COVID-19. However, based on the patient’s clinical improvement, negative PCR results, and resolution of the chest CT in a few days, COVID-19 was ruled out. There was a consistency between CT scan findings and the mechanism and severity of the injury. Therefore the GGO and lung consolidation were felt to be due to trauma to the lung. The patient underwent tracheostomy and was discharged home with a tracheostomy.

#### Case 11

**Figure 11.**
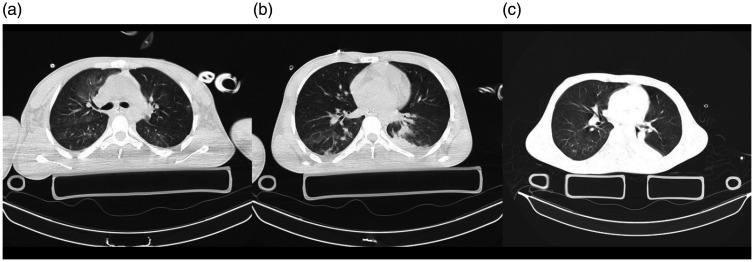
A 20-year-old man with multiple trauma and chest trauma admitted to ICU, who was initially suspicious for COVID-19 too, but based on the improvement of clinical and paraclinical results, COVID-19 was ruled out. 11 A: faint patchy GGO is seen in both lungs more in the anterior aspect of the right upper lobe. 11B: an axial image of the chest CT scan in the level of inferior pulmonary vein showed a small area of consolidation in the posterior aspect of the left lower lobe with minimal adjacent pleural effusion. Mild patchy GGO is seen in the posterior of the right lower lobe. 11 C: there is faint small patchy GGO in the posterior of the right lower lobe. Also, complete atelectasis of the left lower lobe is noted.

A 20-year-old diabetic male was admitted to ICU with intracranial hematoma, base of skull fracture, left side lung contusion, laceration of liver, fracture of femur and T4 vertebrae. The patient had fever, and the initial chest imaging (Figure 11(a)), which showed GGO in the right lung made us suspicious of COVID-19. This was ruled out based on the resolution of chest CT (Figure 11(b) and (c)) and negative PCR results. Though the GGO sign in the CT could be in favor of COVID-19, resolution of this sign after a day can be suggestive of lung contusion on the contralateral side based on countercoup phenomena.

## Discussion

According to the available guidelines, the diagnosis of COVID-19 is based on the initial respiratory symptoms, PCR results, and a chest CT scan in favor of SARS-CoV-2 involvement. However, the diagnosis of pneumonia in trauma patients is not straightforward since the evaluation of respiratory symptoms, such as cough, chest pain, and dyspnea, is not applicable for all cases, especially those with a decreased level of consciousness, trauma to the chest and lung contusion. In addition, many patients in the trauma settings have high levels of inflammatory blood markers, such as ESR (erythrocyte sedimentation rate), CRP (c-reactive protein), LDH (lactate dehydrogenase), and procalcitonin, that make these markers less helpful in the diagnosis of COVID-19 specifically. The presence of fever, respiratory distress, and new infiltrates in a chest radiograph that does not clear with chest physiotherapy can be some of the clinical manifestations.^8^ At the same time, trauma patients are at higher risk of ARDS and multi-organ failure due to pro-inflammatory responses after their injury and the anti-inflammatory response against this pro-inflammatory condition aggravates the ARDS and multi-organ failure.^9^

Performing SARS-CoV-2 RT-PCR can be advantageous in this setting, but, the sensitivity of PCR tests are not high enough to detect all of the COVID-19 cases, or the upper respiratory samples might not have enough viral load to be detected by the RT-PCR kits.^10^ Chest CT scans are additional diagnostic tools that are helpful in diagnosing COVID-19 in suspicious patients.^11^ Previous studies on COVID-19 demonstrated that the most common features on CT images are GGO or mixed GGO and consolidation with a peripheral distribution and a bilateral, multifocal middle, lower lung, and posterior area involvement.^4,12^ Despite negative PCR tests, such CT features in the context of clinical presentation can be suggestive of COVID-19.^13,14^ However, this again can be challenging in patients with chest trauma and lung contusion.

We believe there are three groups of patients that may present to trauma centers with COVID-19:

### Group A

The first group are patients presenting to trauma centers with no chest injuries who have signs and symptoms of respiratory infection suspicious for SARS-CoV-2 infection during their initial assessment. These patients present with respiratory involvement on chest CT scan obtained for the management of their trauma, such as cases 1, 2, 7 and 8 described in this report. They did not have trauma to the chest, but the initial chest CT was abnormal and showed signs of viral pneumonia in favor of SARS-CoV-2 infection.

### Group B

The second group of patients are those with multiple trauma, including chest trauma. However, chest images are also suggestive of viral pneumonia. Differentiating lung contusion from viral pneumonia is crucial in this group of patients, such as patients 3 and 4 in this report. These are the most challenging cases since the assessment of signs and symptoms of COVID-19 is not applicable due to chest trauma, and detecting viral involvement of the lung is difficult because of lung contusion.

Pulmonary contusion is one of the most common lung injuries in chest trauma and usually occurs at the site of the injury, or on the opposite side through countercoup phenomena. The manifestations in the chest CT are patchy airspace opacities and consolidations with non-segmental distribution and subpleural sparing.^15^ Based on our experience, the radiological manifestation of contusion and lung opacities in chest CT scan of trauma patients with incidental COVID-19 pneumonia is relatively similar. Patchy peripheral consolidation and GGO are common findings among both groups. Although subpleural sparing was identified in traumatic patients, which is similar to findings of lung contusion,^16^ some studies reported these findings in COVID 19 pneumonia,^12^ whereas others, not.^17^ Also, round central opacities are usually not seen in the first images of trauma patients unless complicated by nosocomial infections or fat emboli during admission; this pattern was visualized in our patients with COVID-19. Associated findings of pneumothorax, pneumomediastinum, and rib fractures are seen in patients with pulmonary contusion. Moreover, the timing of resolution of the chest image findings can help us to determine the probable cause of these opacities. The resolution of lung contusion starts within 24–48 hours and can be completely clear in a few days^15^ such as case 10 in this report.

### Group C

The third group are patients who do not have any symptoms suggestive of COVID-19 at the time of presentation to the hospital, and their chest imaging is normal. In the first days of hospital admission, they develop respiratory symptoms and a chest CT suggestive of viral pneumonia. Diagnosing COVID-19 in this group can also be challenging when PCR results for COVID-19 are negative. The diagnosis in this group is mostly based on the clinical course of the patient, paraclinical results, and careful evaluation of CT scans. For example, cases 9 and 10 in this report showed signs of respiratory distress, but their chest imaging was clear in a few days, and the imaging manifestations were consistent with their mechanism and severity of the trauma, so COVID-19 was ruled out. On the other hand, case 5 did not have a chest injury or any other mechanism that explained the findings on the CT scan, and the chest CT did not clear gradually; therefore, this case was considered as COVID-19 though the PCR was negative.

## Conclusion

Diagnosis of COVID-19 is challenging in trauma patients, especially among those with chest trauma and lung contusion. Since PCR for SARS-CoV-2 is not always helpful in determining the cause of pneumonia, CT manifestations along with the clinical and paraclinical presentation and course of improvement can be helpful in diagnosing COVID-19.

## Supplemental Material

sj-pdf-1-tra-10.1177_1460408620950602 - Supplemental material for Challenges of diagnosis of COVID-19 in trauma patients: A case series Click here for additional data file. Supplemental material, sj-pdf-1-tra-10.1177_1460408620950602 for Challenges of diagnosis of COVID-19 in trauma patients: A case series by Golnar Sabetian, Farnia Feiz, Alireza Shakibafard, Hossein Abdolrahimzadeh Fard, Sepideh Sefidbakht, Seyed Hamed Jafari, HamidReza Abbasi, Masoomeh Zare, Amir Roudgari, Farid Zand, Mansoor Masjedi and Shahram Paydar in Trauma
