# Supplementary material for: Challenges of diagnosis of COVID-19 in trauma patients: A case series
Source: Trauma. 2021 Jul;23(3):218–29. doi: 10.1177/1460408620950602 (PMC7435208; doi:10.1177/1460408620950602)
Supplement: sj-pdf-1-tra-10.1177_1460408620950602 - Supplemental material for Challenges of diagnosis of COVID-19 in trauma patients: A case series [file sj-pdf-1-tra-10.1177_1460408620950602.pdf]

Appendix:

Table 1. detailed lab data of COVID-19 patients in their hospital course

Lab data	Case 1	Case 2	Case 3	Case 4	Case 5	Case 6	Case 7	Case 8
**Blood cell counts on day 1 of admission**								
**Total WBC[1] count ($\times 10^9$ cells/L)**	15.7	4	17.1	18.6	19	29.3	16	21
Absolute Neutrophil counts ($\times 10^9$ cells/L, fraction %)	13.7 (87.8%)	3.5 (87.8%)	13.8 (80.9%)	15.6 (84%)	16.3 (86%)	25.2 (86%)	8.3 (52%)	16.2 (77%)
**Absolute Lymphocyte count (cells/ml, fraction%)**	847 (5.4%)	228 (5.7%)	1760 (10.3%)	17100 (9.2%)	1254 (6.6%)	1172 (4%)	6720 (42%)	2520 (12%)
**Platelet count** ($\times 10^9$ cells/L**)**	150	100	191	219	287	159	271	257
**Blood cell counts during hospital course**								
**total WBC count ($\times 10^9$ cells/L)**	5.9	2.7	26.9	17.3	24.3	18.5	8.8	N/A
Absolute Neutrophil counts ($\times 10^9$ cells/L, fraction %)	4.6 (78.8%)	1.9 (70%)	2.5 (91.7%)	14.3 (82.5%)	20.2 (83%)	14.4 (78%)	7.4 (84%)	N/A
**Absolute Lymphocyte count (cells/ml, fraction %)**	991 (16.8%)	297 (11%)	2232 (8.3%)	2076 (12%)	2138 (8.8%)	2645 (14.3%)	1434 (16.3%)	N/A
**Platelet count** ($\times 10^9$ cells/L**)**	110	70	303	163	1200	160	158	N/A
**Blood cell counts at the time of death/ discharge**								
**total WBC count ($\times 10^9$ cells/L)**	9.8	3.2	13.8	10.4	17.6	12.1	6.9	N/A
Absolute Neutrophil counts ($\times 10^9$ cells/L, fraction %)	8.2 (83.9%)	2.6 (80%)	11.3 (82.1%)	8.8 (84.9%)	14.1 (80%)	8.6 (71%)	4.1 (58.9%)	N/A
**Absolute Lymphocyte count (cells/ml, fraction %)**	1185 (12.1%)	192 (6%)	2083 (15.1%)	884 (8.5%)	1988 (11.3%)	1923 (15.9%)	2166 (31.4%)	N/A
**Platelet count** ($\times 10^9$ cells/L**)**	24	50	120	200	537	150	204	N/A
**Procalcitonin**	N/A	N/A	N/A	1.39	N/A	1.69	N/A	014
**CRP[2] levels (mg/L)**								
**On day 1 of admission**	150	74	89	8	N/A	92	N/A	60
**In the hospital course**	110	80	81	56	N/A	55	79	N/A

**At the time of death/ discharge**	24	N/A	25	N/A	N/A	N/A	N/A	N/A
**ESR[3] levels (mm/hour)**								
**On day 1 of admission**	N/A	25	34	50	N/A	32	N/A	35
**In the hospital course**	42	25	77	83	NA	68	39	N/A
**At the time of death/ discharge**	38	N/A	14	N/A	N/A	N/A	N/A	N/A
**BUN[4] levels mg/dL:**								
**On day 1 of admission**	22	25	14	8	14	23	7	14
**In the hospital course**	100	40	20	56	34	20	7	N/A
**At the time of death/ discharge**	101	100	19	41	21	15	6	N/A
**Creatinine levels (mg/dL)**								
**On day 1 of admission**	1.15	1.5	0.99	1.01	1.09	0.99	0.4	1.3
**In the hospital course**	3.9	1.7	1.2	1.84	1.24	1.17	0.8	N/A
**At the time of death/ discharge**	4.1	4	1.5	1.1	1.07	0.74	0.6	N/A
**LDH[5]**	1009	800	977	550	600	780	884	450
**SGOT[6] level (U/L)**								
**On day 1 of admission**	70	80	66	73	34	N/A	48	23
**In the hospital course**	18	N/A	22	72	121	216	N/A	N/A
**At the time of death/ discharge**	N/A	N/A	17	126	N/A	79	N/A	N/A
**SGPT[7] levels (U/L)**								
**On day 1 of admission**	46	68	55	12	24	108	27	31
**In the hospital course**	3	N/A	15	77	120	83	N/A	N/A
**At the time of death/ discharge**	N/A	N/A	17	168	N/A	N/A	N/A	N/A
**D-Dimer**	1500	1100	850	3162	1500	2747	N/A	1718
**Troponin**	N/A	N/A	78	449	<1.5	N/A	N/A	N/A

**On day 1 of admission**								
**PaO2[8] mmHg**	209	55	42	30	25.9	140	39.9	49.9
**SaO2[9] %**	93%	87.9%	78%	56.4%	47%	98%	69.3%	82.4%
**In the hospital course**								
**PaO2, mmHg**	58	45	78	62	46.7	35	36.5	N/A
**SaO2, %**	91%	70%	74%	91.8%	69%	64%	88.7%	N/A
**At the time of death/ discharge**								
**PaO2, mmHg**	164	60	N/A	125	68.5	139	153	N/A
**SaO2. %**	98%	67%	56.2%	98.7%	94.9%	99%	19.2%	N/A

[1]WBC: White Blood Cells, [2]CRP: C-Reactive Protein, [3]ESR: Erythrocyte Sedimentation Rate, [4]LDH: Lactate Dehydrogenase, [5]BUN: Blood Urea Nitrogen, [6]SGOT: Serum Glutamic-Oxaloacetic Transaminase, [7]SGPT: Serum Glutamic-Pyruvic Transaminase, [8]PaO2: partial pressure of oxygen, [9]SaO2: Oxygen saturation.

Table 2. detailed lab data of non- COVID-19 cases in their hospital course:

Lab data	Case A	Case B	Case C
**Blood cell counts on day 1 of admission**			
**Total WBC[1] count ($\times 10^9$ cells/L)**	19.3	7	9.7
Absolute Neutrophil counts ($\times 10^9$ cells/L, fraction %)	17.2 (89.2%)	3.4 (49%)	7.7 (79%)
**Absolute Lymphocyte count (cells/ml, fraction%)**	1582 (8.2%)	2653 (37.9%)	1067 (11%)
**Platelet count** ($\times 10^9$ cells/L**)**	217	183	160
**Blood cell counts in the course of hospital**			
**Total WBC count ($\times 10^9$ cells/L)**	21.8	22	13.5
Absolute Neutrophil counts ($\times 10^9$ cells/L, fraction %)	19 (87.5%)	19.1 (87%)	1.1 (84.1%)
**Absolute Lymphocyte count (cells/ml, fraction%)**	1329 (6.1%)	1056 (4.8%)	1242 (9.2%)
**Platelet count** ($\times 10^9$ cells/L**)**	272	238	222

**Blood cell counts at the time of death/ discharge**			
**Total WBC count (×10^9 cells/L)**	6.2	12.8	12.9
Absolute Neutrophil counts (×10^9 cells/L, fraction %)	3.9 (64.3%)	10.5 (82%)	9.8 (76.6%)
**Absolute Lymphocyte count (cells/ml, fraction%)**	1240 (20.9%)	1075 (8.4%)	1496 (11.6%)
**Platelet count** (×10^9 cells/L**)**	268	369	317
**Procalcitonin**	0.78	0.5	4.29
**CRP[2] levels (mg/L)**			
**On day 1 of admission**	N/A	N/A	48
**In the hospital course**	80	99	N/A
**At the time of death/ discharge**	N/A	66	N/A
**ESR[3] levels (mm/hour)**			
**On day 1 of admission**	N/A	N/A	70
**In the hospital course**	81	29	N/A
**At the time of death/ discharge**	N/A	81	N/A
**BUN[4] levels mg/dL**			
**On day 1 of admission**	9	16	25
**In the hospital course**	11	12	31
**At the time of death/ discharge**	12	14	15
**Creatinine levels (mg/dL)**			
**On day 1 of admission**	1.1	0.8	0.8
**In the hospital course**	1.2	0.7	0.9
**At the time of death/ discharge**	0.7	0.8	0.5
**LDH[5]**	N/A	N/A	1433
**SGOT level (U/L)**			
**On day 1 of admission**	75	44	234
**In the hospital course**	77	81	59
**At the time of death/ discharge**	N/A	N/A	N/A
**SGPT[6] level (U/L)**			
**On day 1 of admission**	21	27	175

**In the hospital course**	42	56	83
**At the time of death/ discharge**	N/A	N/A	N/A
**D-Dimer**	4077	3062	N/A
**Troponin**	N/A	1035	N/A
**On day 1 of admission**			
**PaO2[7]**	41.5	105	55.2
**SaO2[8]**	74.2%	97.4%	88%
**In the hospital course**			
**PaO2**	41.5	65	N/A
**SaO2**	74.2%	71.3%	N/A
**At the time of death/ discharge**			
**PaO2**	168.7	136	140
**SaO2**	99.2%	99%	N/A

[1]WBC: White Blood Cells, [2]CRP: C-Reactive Protein, [3]ESR: Erythrocyte Sedimentation Rate, [4]LDH: Lactate Dehydrogenase, [5]BUN: Blood Urea Nitrogen, [6]SGOT: Serum Glutamic-Oxaloacetic Transaminase, [7]SGPT: Serum Glutamic-Pyruvic Transaminase, [8]PaO2: partial pressure of oxygen, [9]SaO2: Oxygen saturation.
